# Induction of immune response in chickens primed in ovo with an inactivated H9N2 avian influenza virus vaccine

**DOI:** 10.1186/s13104-018-3537-9

**Published:** 2018-07-03

**Authors:** Jake Astill, Tamiru Alkie, Alexander Yitbarek, Khaled Taha-Abdelaziz, Jegarubee Bavananthasivam, Éva Nagy, James John Petrik, Shayan Sharif

**Affiliations:** 1 Department of Pathobiology, Ontario Veterinary College, University of Guelph, Guelph, ON N1G 2W1 Canada; 2 Present Address: Department of Biology, Wilfred Laurier University, Waterloo, ON N2L 3C5 Canada; 3 Pathology Department, Faculty of Veterinary Medicine, Beni-Suef University, Al Shamlah, Beni-Suef, 62511 Egypt; 4 Department of Biomedical Sciences, Ontario Veterinary College, University of Guelph, Guelph, ON N1G 2W1 Canada

**Keywords:** Antibody, Beta-propiolactone, Cell-mediated, CpG ODN, H9N2 avian influenza virus, In ovo, Intramuscular, Toll-like receptor 21, Vaccine

## Abstract

**Objective:**

Infection of chickens with low pathogenic avian influenza virus, such as H9N2 virus, culminates in decreased egg production and increased mortality and morbidity if co-infection with other respiratory pathogens occurs. We have previously observed the induction of antibody- and cell-mediated immune responses after intramuscular administration of an H9N2 beta-propiolactone inactivated virus vaccine to chickens. Given the fact that in ovo vaccination represents a practical option for vaccination against H9N2 AIV in chickens, in the current study, we set out to characterize immune responses in chickens against a beta-propiolactone inactivated H9N2 virus vaccine after primary vaccination in ovo on embryonic day 18, and secondary intramuscular vaccination on day 14 post-hatch. We also included the Toll-like receptor 21 ligand, CpG ODN 2007, and an oil emulsion adjuvant, AddaVax™, as adjuvants for the vaccines.

**Results:**

Antibody-mediated immune responses were observed after administering the secondary intramuscular vaccine. Cell-mediated immune responses were observed in chickens that received the beta-propiolactone inactivated H9N2 virus combined with AddaVax™. Our results demonstrate that adaptive immune responses can be induced in chickens after a primary in ovo vaccination and secondary intramuscular vaccination.

## Introduction

Avian influenza virus (AIV) H9N2 subtype is an enveloped, negative-sense single-stranded segmented RNA virus in the family *Orthomyxoviridae* that is now the most widespread AIV subtype found in poultry [1]. During infection, chickens are often free of clinical signs, but infection can increase morbidity and mortality during co-infection with other respiratory pathogens [2]. Poultry vaccination against H9N2 virus is a possible way to decrease infection and transmission among birds. In ovo administration is a potential vaccination route for chickens, as technology already exists that can facilitate mass vaccinations, and commercial in ovo vaccines against various pathogens including Marek’s disease virus (MDV) already exist [3].

In ovo vaccines are typically delivered on embryonic day 18 (ED18) to the amniotic cavity, during this time the embryo is typically imbibing amniotic fluid [3]. Early research demonstrated that administration of an inactivated H5N9 AIV vaccine in ovo resulted in post-hatch (ph) seroconversion and induction of immune responses in hatched chicks against H5N9 virus [4]. Various other types of AIV vaccines have been studied for in ovo administration, including; non-replicating adenovirus vectors expressing hemagglutinin (HA) proteins [5–7], attenuated vaccines [8], and recombinant attenuated vaccines [9]. Attenuated viral vaccines generally induce strong immune responses, however there is always the risk of mutation back to the virulent form [10]. Vector virus vaccines for influenza virus can lead to the induction of immune responses against the HA protein, however, internal influenza proteins in the virion are absent in the vaccine, and peptides from these proteins are often the target of cell-mediated immune responses that can be cross-protective to other subtypes of AIV [11].

In chickens, Toll-like receptor (TLR) ligands have adjuvant capabilities when combined with inactivated AIV vaccines administered in vivo [12–14]. CpG ODNs have also been shown to be immunostimulatory when administered to the chicken embryo. In ovo administration of CpG ODN has been shown to decrease in ovo replication of H4N6 AIV and infectious bronchitis virus [15, 16], in addition to having protective effects following experimental challenge with *Salmonella* and infectious laryngotracheitis virus ph [17, 18]. In the present study, embryos were vaccinated in ovo with H9N2 beta-propiolactone (BPL) whole inactivated virus (WIV) vaccines, followed by a secondary intramuscular (IM) vaccination ph. Subsequently, cell- and antibody-mediated immune responses were quantified in chickens. CpG ODN 2007 was also included as an adjuvant in this study to determine its adjuvant effects when administered in ovo.

## Main text

### Materials and methods

#### Hatching and housing of chickens

Sixty-eight specific pathogen free (SPF) eggs were purchased from the Canadian Food Inspection Agency (CFIA) (Ottawa, Canada) and were incubated at Arkell Poultry Research Station (University of Guelph, Ontario, Canada). Hatched chicks were housed at the isolation facility at the Ontario Veterinary College at the University of Guelph.

#### BPL inactivation of H9N2 virus

H9N2 AIV (A/Turkey/Wisconsin/1/66) was propagated in SPF chicken eggs. Thirty-eight parts allantoic fluid were combined with one part 0.5 M disodium phosphate (DSP), then a 4% BPL solution was mixed with above solution to produce a 0.1% BPL solution. The mixture was incubated for 30 min on ice, followed by 2 h of incubation at 37 °C to hydrolyze the remaining BPL. The pH of the solution was then adjusted to ~ 7.4 with 7% sodium bicarbonate. Concentration of the inactivated virus and determination of protein concentration was performed as previously described [14].

#### Experiment design and vaccine groups

On ED18, 17 live embryos per group were vaccinated in ovo via the amniotic cavity using a one inch long 25 gauge needle. Embryos in three groups received 15 µg of BPL inactivated H9N2 virus, one received it alone (BPL), one received it combined with an oil emulsion adjuvant, AddaVax™ (BPL + Add), and one received it combined with 2 µg of CpG ODN 2007 (BPL + CpG). CpG ODN 2007 was purchased from Invivogen (San Diego, California, USA). Hatchability ranged from 88 to 100%, and for each group, 15 chicks continued in the experiment for subsequent sample collection and treatment. On day 14 ph chickens received a secondary IM vaccine (identical to the first vaccine) in the thigh muscle. One group received just phosphate buffered saline for both vaccinations (PBS).

#### Hemagglutination inhibition (HI) and enzyme-linked immunosorbent assay (ELISA)

Methods for the ELISA and HI assay are described previously [14]. For the ELISA, serum was diluted and relative antibody titer was calculated in relation to a serially diluted high titer serum sample using methods described previously [19].

#### Analysis of cell-mediated immune responses

Ten days after the secondary vaccination spleens were collected from 5 chickens per group. Chickens were euthanized humanely via carbon dioxide inhalation. Splenocytes were isolated and seeded in 48 well plates and stimulated with 1 μg/ml BPL inactivated H9N2 virus as described previously [12]. At 12 and 24 h post-stimulation, cells were collected for analysis of cytokine gene expression and at 48 and 72 h, supernatants were collected to assess interferon (IFN)-γ production using a chicken IFN-γ Cytoset ELISA kit (Invitrogen™) following manufacturer’s instructions.

#### Gene expression analysis

RNA extraction and cDNA synthesis methods used have been previously described [12]. Real-time relative expression of each gene was calculated relative to chicken β-actin, using Light-Cycler^®^ 480 II (Roche Diagnostics GmbH) software, as described previously [20]. Primer sequences for genes; IL-2, β-actin, and IFN-γ can be found in [21–23], respectively.

#### Statistical analysis

All data, including antibody titers and cytokine expression and production levels were compared using Duncan’s Multiple Range Test using R© software. When *p *< 0.05, differences in means were considered significant.

### Results

#### Antibody-mediated immune responses

**Fig. 1:**
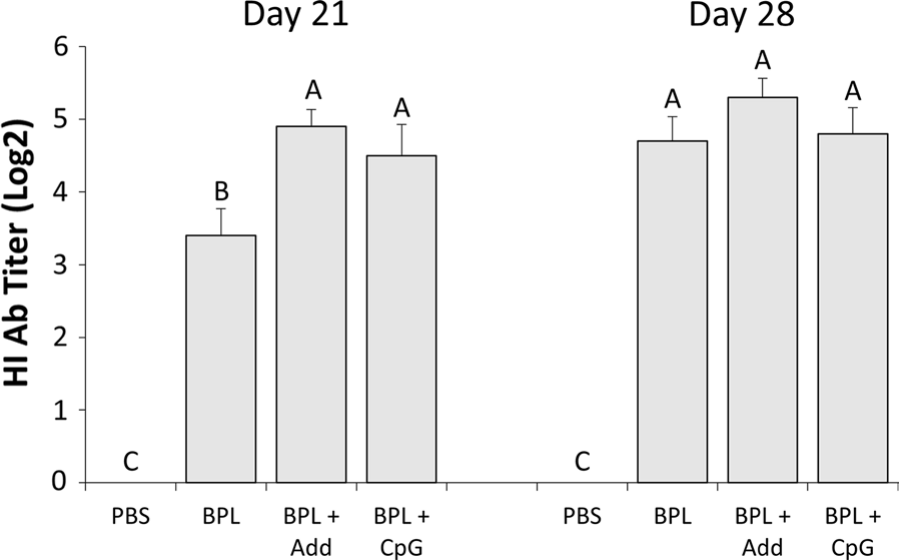
HI titers in serum against H9N2 AIV. Average serum HI titers 21 and 28 days ph, from 10 chickens per group. Chickens were vaccinated in ovo on ED18 and received a second vaccination 14 days ph. Vaccines consisted of 15 µg of BPL inactivated H9N2 virus administered alone (BPL), with AddaVax™ (BPL + Add), or with 2 µg CpG ODN 2007 (BPL + CpG). One group received just PBS as a negative control (PBS). Serum was collected weekly following hatch. HI titers were first observed in serum 7 days post secondary vaccination. Group means that share the same letter did not differ significantly. Standard error of the mean is indicated with error bars. Data were analyzed using Duncan’s Multiple Range Test and differences in means were considered significant if *p* < 0.05

**Fig. 2:**
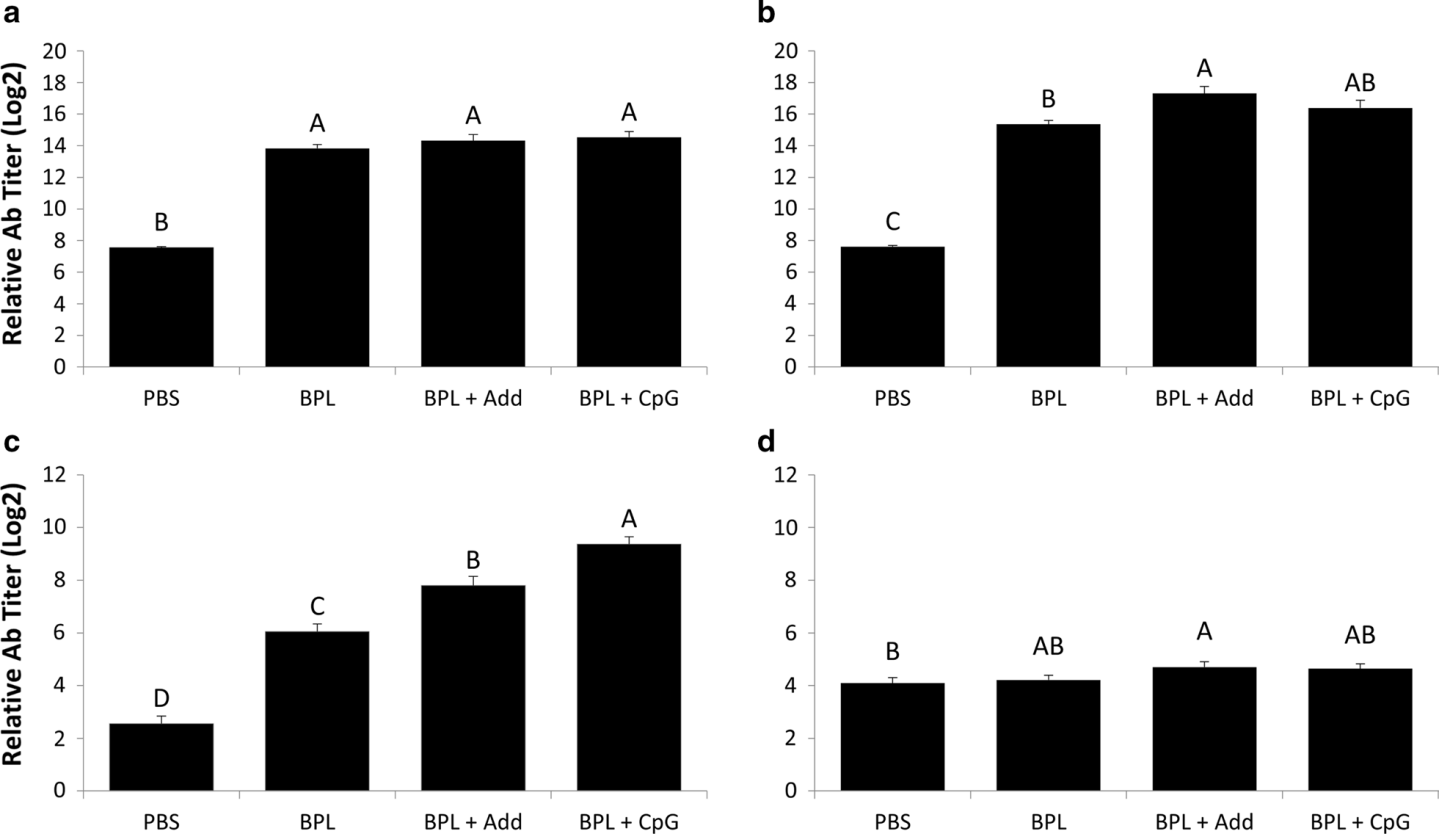
IgY and IgM titers in serum against H9N2 AIV. Average serum IgY titers **a** 21 days and **b** 28 days ph and average serum IgM titers **c** 21 days and **d** 28 days ph, from 10 chickens per group. Chickens were vaccinated in ovo on ED18 and received a second vaccination 14 days ph. Vaccines consisted of 15 µg of BPL inactivated H9N2 virus administered alone (BPL), with AddaVax™ (BPL + Add), or with 2 µg CpG ODN 2007 (BPL + CpG). One group received just PBS as a negative control (PBS). Serum was collected weekly following hatch. Antibody titers were first observed in serum 7 days post secondary vaccination. Group means that share the same letter did not differ significantly. Standard error of the mean is indicated with error bars. Data were analyzed using Duncan’s Multiple Range Test and differences in means were considered significant if *p* < 0.05

The first evidence of antibody responses occurred on day 21 ph (day 7 post-secondary vaccination), and all chickens that received a vaccine demonstrated the presence of antibody-mediated immune responses compared to none in the PBS group. There were significant differences in HI titers between vaccinated groups on day 21 ph, as both the BPL + Add and BPL + CpG vaccines resulted in 2-fold higher HI titers compared to the BPL vaccine (*p* < 0.05) (Fig. 1). HI titers in chickens that received the BPL vaccine demonstrated a significant increase (*p *< 0.05) from day 21 to 28 ph, but there were no significant differences evident between vaccinated groups on day 28 ph (Fig. 1). Serum IgY titers were also first seen on day 21 ph, although there were no significant differences in IgY titers at this time point between vaccinated groups (Fig. 2a). However, on day 28 ph, serum IgY titers were significantly increased in chickens that received the BPL + Add vaccine relative to the BPL vaccine (*p* < 0.01) (Fig. 2b). IgM titers were also first evident on day 21 ph. The BPL + CpG vaccine induced significantly higher IgM titers that were 3-fold and 10-fold higher than those induced by the BPL + Add and BPL vaccines, respectively (*p* < 0.01) (Fig. 2c). Also, on day 21 ph the BPL + Add vaccine induced 3-fold significantly higher serum IgM titers than the BPL vaccine (*p* < 0.01) (Fig. 2c). By day 28 ph, serum IgM titers had decreased dramatically, and only the chickens that received the BPL + Add vaccine demonstrated serum IgM titers that were significantly higher than the PBS control group (*p* < 0.05) (Fig. 2d).

#### Cell-mediated immune responses

**Fig. 3:**
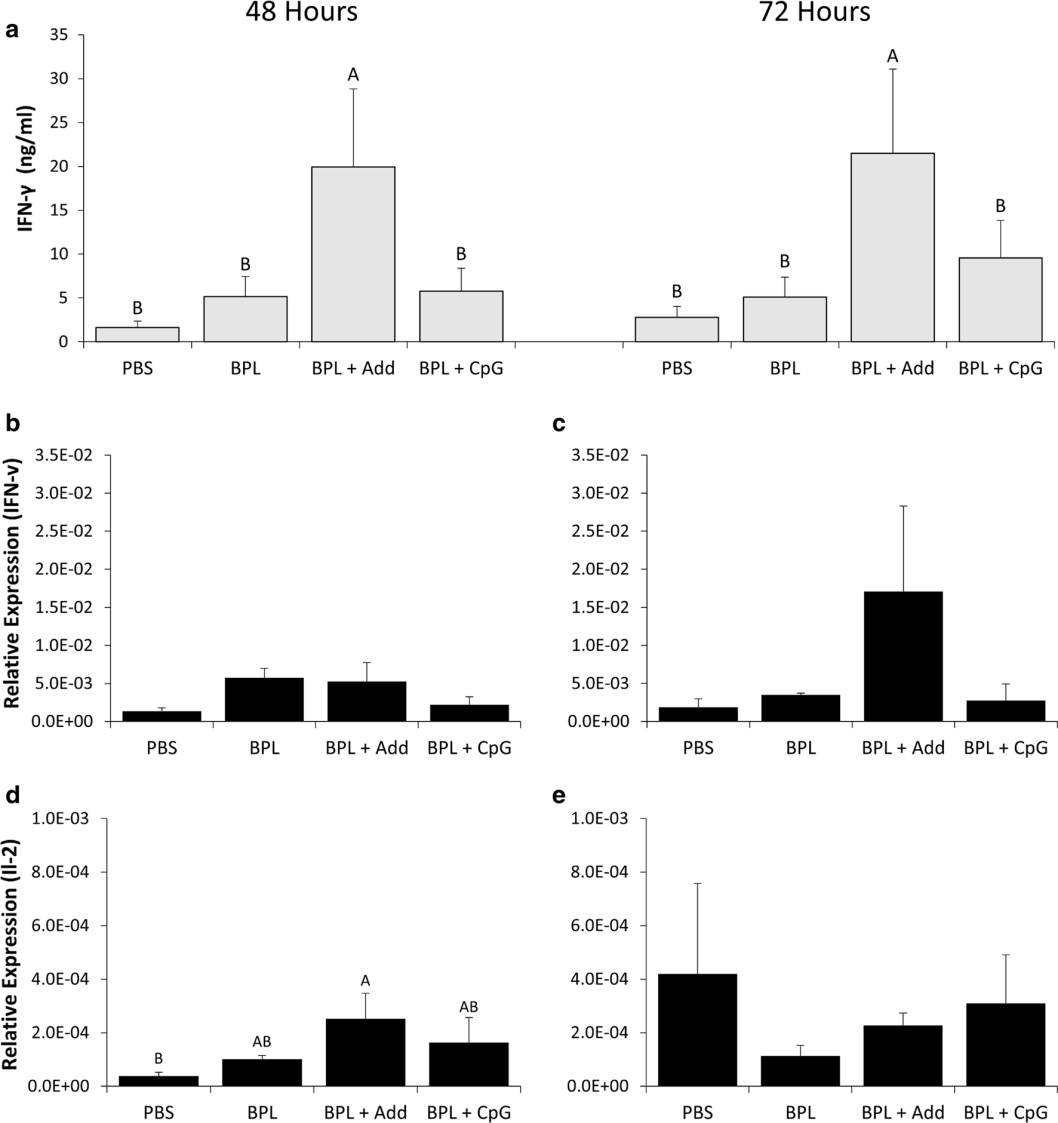
IFN-γ production in stimulated splenocyte supernatants and IFN-γ and IL-2 expression in stimulated splenocytes. Chickens were vaccinated in ovo on ED18 and received a second vaccination 14 days ph. Vaccines consisted of 15 µg of BPL inactivated H9N2 virus administered alone (BPL), with AddaVax™ (BPL + Add), or with 2 µg CpG ODN 2007 (BPL + CpG). One group received just PBS as a negative control (PBS). Five spleens from 5 birds per group were collected 10 days post secondary vaccination. Splenocytes were isolated and seeded in 48 well plates and stimulated with 1 μg/ml of BPL inactivated H9N2 virus. Supernatants were collected **a** 48 and 72 h after stimulation and supernatant IFN-γ concentrations were quantified. IFN-γ expression in splenocytes was quantified at **b** 12 h and **c** 24 h post-stimulation and IL-2 expression was quantified at **d** 12 h and **e** 24 h post-stimulation. Gene expression was quantified relative to β-actin expression. Group means that share the same letter did not differ significantly. Standard error of the mean is indicated with error bars. Data was analyzed using Duncan’s Multiple Range Test and differences in means were considered significant if *p* < 0.05

IFN-γ production in supernatants of stimulated splenocytes was assessed at 48 and 72 h post-stimulation. At both time points, significantly more IFN-γ production (4-fold higher) was observed from splenocytes from chickens that received the BPL + Add vaccine compared to all other groups (*p* < 0.05) (Fig. 3a). Gene expression analysis indicated that at 12 h post-stimulation, there were no significant differences in IFN-γ expression (Fig. 3b). By 24 h post-stimulation, the highest level of IFN-γ expression occurred in splenocytes from chickens that received the BPL + Add vaccine, although this difference was not significant (Fig. 3c). Lastly, expression of interleukin (IL)-2 was also quantified in splenocytes 12 and 24 h post-stimulation. We observed a significant increase in expression of IL-2 in splenocytes from chickens that received the BPL + Add vaccine, relative to splenocytes from chickens that received the BPL vaccine or PBS (2- and 6-fold greater, respectively) (*p* < 0.05) (Fig. 3d). At 24 h post-stimulation there were no significant differences in IL-2 expression (Fig. 3e).

### Discussion

There is little data on in ovo whole inactivated virus (WIV) influenza vaccines, however our findings concerning the induction of antibody-mediated immune responses contrast with some of the previously published results. Previously, a group vaccinated ED18 embryos with BPL inactivated H5N9 AIV and observed serum HI titers in chickens by 4 weeks of age [4], however it is difficult to compare the dose of inactivated influenza virus administered between studies, due to differences in dose quantification. In ovo vaccine antigen dose appears to be important for the induction of antibody responses, and this relationship has been demonstrated for in ovo administered adenovirus vector vaccines expressing HA proteins [5–7]. Additionally, the time for antibody responses to appear in hatched chickens following in ovo vaccination seems to take longer compared to vaccines administered ph in chickens. For example, in a study where chicken embryos were administered 10^9^ infectious units of an adenovirus vector influenza vaccine, less than one-percent of chickens displayed serum HI titers by 10 days ph [6]. By 20 and 40 days ph, the percentage rose to 65 and 85%, respectively; notably, this dose was the highest in the study and a 10-fold lower dose displayed a similar trend but at lower magnitudes. This suggests that our 14 day time frame prior to the second vaccine could have been too short to detect antibodies induced by in ovo vaccination alone.

Past research has shown that CpG ODN 2007 can increase systemic antibody titers in chickens when combined with a formalin inactivated H9N2 virus vaccine administered intramuscularly [14]. We have also demonstrated that CpG ODN 2007 has the same effects on antibody response when combined with a BPL inactivated H9N2 virus vaccine [24]. In the present study, consistent differences in serum IgY and HI titers were not evident when comparing non-adjuvanted to CpG ODN 2007-adjuvanted vaccines. Nevertheless, following the secondary IM vaccination, serum IgM titers were the highest in chickens that received vaccines with CpG ODN 2007 as an adjuvant. This further demonstrates the adjuvant capabilities of CpG ODN 2007 as an adjuvant for inactivated IM influenza vaccines.

Cell mediated-immune responses have been demonstrated to exhibit protective responses against AIV in chickens following in ovo vaccination. For example, in a study where chicken embryos were vaccinated in ovo with a recombinant attenuated H5N1 vaccine, only 30% of chickens developed antibodies to the H5 protein, yet 80% of vaccinated chickens survived a lethal challenge, compared to no survival in the control group [9]. Although not experimentally demonstrated, the enhanced survival was partially attributed to cell-mediated immune responses. Our observations suggest that BPL inactivated H9N2 virus vaccines can induce cell-mediated immune responses after a primary in ovo and secondary IM vaccination. Research in mice has suggested that influenza BPL WIV vaccines induce cell-mediated immune responses, specifically CD8+ T cell responses, because of structural characteristics that BPL inactivated influenza viruses possess [25]. Using IM vaccination, our group made a similar finding in chickens [24], however we only observed cell-mediated immune responses when CpG ODN 2007 was combined with the BPL WIV vaccine. Strikingly, CpG ODN 2007 did not enhance cell-mediated immune responses in the present study. There is ample evidence that demonstrates that CpG ODNs are immunostimulatory when administered to chicken embryos [15–18]. Despite this, our results suggest that CpG ODN 2007 may not be suited to induce cell-mediated immune responses as an in ovo vaccine adjuvant, however different doses should be studied. The oil emulsion adjuvant used in the study, Addavax™, induced modest increases in antibody titers in addition to inducing cell-mediated immune responses, suggesting it to be an effective adjuvant for in ovo vaccination, but future studies should examine this further.

In conclusion, the present study has demonstrated the immune responses induced in chickens after a primary in ovo and secondary IM vaccination with a BPL WIV H9N2 vaccine. Future studies should determine if greater doses of H9N2 WIV vaccines administered in ovo can induce serum antibody responses.

## Limitations

Antibodies were not detected in serum until after secondary vaccination. Had more time been designated to detecting responses from in ovo vaccination alone, perhaps these responses would have been observed.

## Abbreviations

AIV: avian influenza virus
IM: intramuscular
BPL: beta-propiolactone
WIV: whole inactivated virus
TLR: Toll-like receptor
MDV: Marek’s disease virus
ED18: embryonic day 18
IFN: interferon
HI: hemagglutination inhibition
IL: interleukin
ph: post-hatch
